# Sleep quality and associated factors during the COVID-19 epidemic among community non-medical anti-epidemic Workers of Wuhan, China

**DOI:** 10.1186/s12889-021-11312-8

**Published:** 2021-06-30

**Authors:** Guanglin Si, Yi Xu, Mengying Li, Yuting Zhang, Shuzhen Peng, Xiaodong Tan

**Affiliations:** 1 School of Health Sciences, Wuhan University, Wuhan, 430071 China; 2 School of Nursing, Shenzhen University, Shenzhen, 518037 China; 3 Huangpi District People’s Hospital, Wuhan, 430300 China; 4 Wuchang University of Technology, Wuhan, 430223 China

**Keywords:** Sleep quality, Community non-medical anti-epidemic workers, COVID-19, Wuhan

## Abstract

**Background:**

Since the outbreak of Coronavirus Disease 2019 (COVID-19) in December 2019, community non-medical anti-epidemic workers have played an important role in the prevention of COVID-19 in China. The present study aimed to assess sleep quality and its associated factors among community non-medical anti-epidemic workers.

**Method:**

A survey was conducted using anonymous online questionnaire to collect information from 16 March 2020 to 24 March 2020. A total of 474 participants were included, with a 94.23% completion rate. The questionnaire contained demographic data, physical symptoms, and contact history with COVID-19. The researchers assessed perceived social support by the Multidimensional Scale of Perceived Social Support (MSPSS), assessed perceived stress by the Perceived Stress Scale (PSS), and measured sleep quality by the Pittsburgh Sleep Quality Index (PSQI) questionnaire.

**Results:**

Among the participants, 46.20% reported poor sleep quality. A binary logistic regression revealed that having educational background of junior college or above, being a member of the police force, having contacted individuals with confirmed or suspected COVID-19 infection, having chronic disease(s), having illness within 2 weeks, and having high or moderate perceived stress were significant factors associated with an increased risk of poor sleep quality.

**Conclusion:**

Demographic factors, physical symptoms, history of contact with COVID-19, and perceived stress are significantly associated with poor sleep quality of community non-medical anti-epidemic workers. Thus, targeting these factors might be helpful in enhancing sleep quality of community workers.

## Background

Since the outbreak of Coronavirus Disease 2019 (COVID-19) in December 2019, around the world, the disease’s social consequences of mass confinement at home have caused much stress [1, 2]. The community anti-epidemic workers, both medical and non-medical workers, have played an important role in the community-based prevention of COVID-19 in China. The non-medical workers mainly participated in “population mobility control” and “community digital management”. For example, the workers collected basic information about residents in a door-to-door way, shifted patients with restrictions on the access to each community, and delivered living supplies to residents who were home-quarantined [3, 4]. However, in accordance with the reports on anti-epidemic nurses during COVID-19 and MERS-CoV [5, 6], continuous working under stressful circumstances for many hours and managing potential health risks could have a major influence on the mental health and sleep quality of non-medical anti-epidemic workers [7].

The existing literature regarding associations among individuals’ stress, social support, and health outcomes indicates the potential influence of moderating factors [8]. The community non-medical anti-epidemic workers are exposed to working environments with low resources and high working demands, high stress, and greater physical and psychological stress, which can adversely affect their health status and sleep quality. Sleep is an important determinant of health, and it is essential for the maintenance of physical and mental health [9]. Sleep disturbance can lead to poor concentration and finances [10] and may increase individuals’ morbidity and mortality [11]. Good sleep quality can help improve immunity against viral infection [12]. Therefore, it is important for researchers to study modifiable factors that are associated with sleep quality.

Recent studies on the epidemic of COVID-19 have been concentrated on clinical epidemiology, prevention, and treatment [13–15]. Few researchers have investigated the sleep quality and other mental-health related issues of community non-medical anti-epidemic workers during the epidemic of COVID-19. Therefore, we conducted the present study to close this gap and better understand sleep quality among community non-medical anti-epidemic workers and relevant factors in Wuhan, China.

## Method

### Participants and procedure

The survey was conducted from 16 March 2020 to 24 March 2020 by anonymous online questionnaires. The convenience-sampling method was used. The researchers randomly selected 15 communities in Wuhan, China. Considering the scale of each community, the researchers selected 30 members for each. The sample size was amplified according to the inefficiency of 10%, and the final expected sample size was 495. The inclusion criteria and exclusion criteria were as follows: (a) Inclusion criteria: (1) The worker had at least 1 month’s experience in anti-epidemic work. (2) The worker had not suffered from mental illness and had not been stimulated by major adverse life events. (3) The worker was willing to participate in the survey. (b) Exclusion criteria: (1) The worker had less than 1 month’s experience in anti-epidemic work. (2) The worker was a shift worker. (3) The worker was unwilling to participate in the survey.

A total of 503 community non-medical anti-epidemic workers in Wuhan, China, participated in the survey. After excluding the questionnaires with repeated filling (ID and basic information are completely consistent) or suspected false answers, the study finally included a total of 474 valid samples, with a 94.23% completion rate.

### Instrument

The structured questionnaire comprised of six areas: (1) Demographic variables, including sex, age, educational background, marital status, occupation, and work experience. (2) Contact with an individual with confirmed or suspected COVID-19 infection. (3) Health-related factors, including chronic disease and illness within 2 weeks. Chronic disease was evaluated through the participant’s response (“yes” or “no”) to the question “if you have been diagnosed with diabetes, hypertension, heart disease, arthritis, migraine, asthma, thyroid disease, heart disease, thrombosis, bronchitis/emphysema, osteoporosis, cancer, stomach/peptic ulcer, cerebrovascular disease or other major physical diseases”, which was similar to what Scott described in a previous study [16]. The present researchers defined illness within 2 weeks as acute sickness lasting for the past 2 weeks. (4) Perceived social support, measured by the Multidimensional Scale of Perceived Social Support (MSPSS), which was developed by Zimet et al. in 1990 [17]. The MSPSS scale comprised of 12 items, which are rated on a seven-point scale (1 = very strongly disagree, 7 = very strongly agree). The total perceived social support score was obtained by summing the responses to each item, and a total MSPSS score < 32 indicated serious perceived social support problems, a score ≥ 32 and < 50 indicated some perceived social support problems, and a score > 50 indicated no perceived social support problem. The Chinese version of the MSPSS has demonstrated good reliability and validity [18]. (5) Perceived stress, measured by the Perceived Stress Scale (PSS), which was developed by Cohen et al. in 1983 [19]. The PSS scale comprised of 10 items with a five-point rating scale (0 = never, 4 = always), and the total score of PSS ranged from 0 to 40. A total PSS score > 19 indicated high perceived stress, a total score > 13 and ≤ 19 indicated moderate perceived stress, and a total score ≤ 13 indicated low perceived stress. Previous researchers had examined the Chinese version of the PSS and documented favorable results [20]. (6) Sleep quality, measured by the Pittsburgh Sleep Quality Index (PSQI) questionnaire, which was developed by Buysse et al. in 1989 [21]. The PSQI scale comprised of seven dimensions: subjective sleep quality, sleep latency, sleep duration, habitual sleep efficiency, sleep disturbance, use of sleep medication, and daytime dysfunction. Each dimension was scored on a scale from 0 to 3, with a total score ranging from 0 to 21. The Chinese version of the PSQI has good reliability and validity in the Chinese population [22]. According to a previous study [21], a total PSQI score ≤ 5 indicated good sleep quality, and a total score > 5 indicated poor sleep quality.

### Statistical analysis

The present researchers conducted all data analysis by using SPSS version 20.0 (IBM Corp., Armonk, NY, USA). Data were presented by frequencies and percentages. Chi-squared tests were used to examine the rate of poor sleep quality in different groups and to choose independent variables for a binary logistic regression analysis. Independent variables with *p* < 0.05 were included in the binary logistic regression analysis (Forward: LR). Finally, the present study identified variables that had a significant association with poor sleep quality on the basis of odds ratio (OR) and 95% confidence interval (95% CI). The independent variables included sex, age category, marital status, educational background, occupation, work experience, contact with individuals with confirmed or suspected COVID-19 infection, chronic disease, illness within 2 weeks, perceived social support, and perceived stress. The dependent variable was sleep quality. All variables were treated as categorical factors, and *p* < 0.05 (two-sides) was considered as statistically significant.

## Results

### Participant characteristics

Of the participants, 63.71% were male, and 36.29% were female. Ages ranged from 20 to 65 years with an average of 38.94 ± 10.18 years. The percentage of participants whose educational background was junior college or above was 73.42%. The percentage of participants who were married was 68.14%. The percentage of participants who had at least 10 years of work experience was 35.44%. A majority of participants were in the police force (81.01%). A majority of participants did not report a problem with social support (83.76%). Almost half of the participants (48.95%) perceived their stress level as low. Furthermore, the percentage of participants who had contact with the individuals with confirmed or suspected COVID-19 infection was only 16.24%. About a quarter of the participants had one or more chronic diseases (25.53%). Less than a third of the participants had an illness within 2 weeks (28.06%) (Table 1).

**Table 1:** Comparison of sleep quality among participants of different groups (*n* = 474)

Category	Group	Good Rate (n/%)	Poor Rate (n/%)	***p***
**Sex**	male	159 (52.65)	143 (47.35)	0.506
	female	96 (55.81)	76 (44.19)	
**Age category (years)**	20–35	105 (54.69)	87 (45.31)	0.489
	36–50	120 (55.05)	98 (44.95)	
	> 50	30 (46.88)	34 (53.13)	
**Marital status**	unmarried	64 (52.89)	57 (47.11)	0.932
	married	174 (53.87)	149 (46.13)	
	divorced/widowed	17 (56.67)	13 (43.33)	
**Educational background**	junior middle school or below	13 (65.00)	6 (35.00)	0.000
	high school/technical secondary school	74 (69.16)	33 (30.84)	
	junior college or above	168 (48.28)	180 (51.72)	
**Occupation**	community workers	11 (61.11)	7 (38.89)	0.021
	polices	195 (50.78)	189 (49.22)	
	volunteers	49 (68.06)	23 (31.94)	
**Work experience (years)**	≤3	94 (57.67)	69 (42.33)	0.037
	4–6	43 (53.75)	37 (46.25)	
	7–9	41 (65.79)	22 (34.92)	
	≥10	77 (45.83)	91 (54.17)	
**Contact with individuals with confirmed or suspected COVID-19 infection**	yes	29 (37.66)	48 (62.34)	0.002
	no	226 (56.93)	171 (43.07)	
**Chronic disease**	yes	46 (30.02)	75 (61.98)	0.000
	no	209 (59.21)	144 (40.79)	
**Illness within 2 weeks**	yes	39 (29.32)	94 (70.68)	0.000
	no	216 (63.34)	125 (36.66)	
**Perceived Social support**	serious problems	3 (50.00)	3 (50.00)	0.053
	some problems	29 (40.85)	42 (59.15)	
	no problem	223 (56.17)	174 (43.83)	
**Perceived stress**	high	27 (25.00)	81 (75.00)	0.000
	moderate	56 (41.79)	78 (58.21)	
	low	172 (74.14)	60 (25.86)	

### Group comparison of sleep quality

The results of the chi-square test are shown in Table 1. Educational background (*p* = 0.000), occupation (*p* = 0.021), work experience (*p* = 0.037), contact with individuals with confirmed or suspected COVID-19 infection(*p* = 0.002), chronic disease (*p* = 0.000), illness within 2 weeks (*p* = 0.000), and perceived stress (*p* = 0.000) were all associated with sleep quality; participants with educational background of junior college or above, members of the police force, participants with at least 10 years of work experience, participants who had contacted with individuals with confirmed or COVID-19 infection, participants who had chronic disease, participants who had been ill within 2 weeks, and participants with high perceived stress were more likely to report poor sleep quality.

### Factors associated with sleep quality

The present researchers included all significant variables in the binary logistic regression analysis. As Table 2 shows, after adjusting for education level, occupation, work experience, contact with an individual with confirmed or suspected COVID-19 infection, chronic disease, illness within 2 weeks, and perceived stress, the present researchers found that the participants with educational background of junior college or above were 2.38 times more likely to report poor sleep quality than those with educational background of high school or technical secondary school (*p* < 0.01). Compared with volunteers, the members of the police force were 2.07 times more likely to report poor sleep quality (*p* < 0.01). Participants with at least 10 years of work experience were 1.61 times more likely to report poor sleep quality than those with ≤3 years of work experience (*p* < 0.05), and they were 2.22 times more likely to report poor sleep quality than those with 7–9 years of work experience (*p* < 0.05). Participants who had contacted with an individual with confirmed or suspected COVID-19 infection were 2.19 times more likely to report poor sleep quality as those who had not (*p* < 0.01). Participants who had chronic disease were 2.37 times more likely to report poor sleep quality than those who did not (*p* < 0.01). Participants who had been ill within 2 weeks were 4.17 times more likely to report poor sleep quality than those who had not (*p* < 0.01). Compared with the participants with low perceived stress, those with high perceived stress were 8.60 times more likely to report poor sleep quality (*p* < 0.01), and those with moderate perceived stress were 3.99 times more likely to report poor sleep quality(*p* < 0.01).

**Table 2:** Binary logistic regression analysis on factors associated with poor sleep quality (*n =* 474)

Variables	S.E.	OR	95%CI
**Education background (junior college and above as reference)**			
Junior middle school or below	0.51	0.43	0.16–1.16
High school/technical secondary school	0.24	0.42	0.26–0.66**
**Occupation (volunteers as reference)**			
community workers	0.55	1.36	0.47–3.95
polices	0.27	2.07	1.21–3.52**
**Work experience (years) (≥10 as reference)**			
≤ 3	0.22	0.62	0.40–0.96*
4–6	0.27	0.73	0.43–1.24
7–9	0.31	0.45	0.25–0.83*
**Contact with individuals with confirmed or suspected COVID-19 infection (no as reference)**			
Yes	0.26	2.19	1.32–3.61**
**Chronic disease (no as reference)**			
Yes	0.22	2.37	1.55–3.62**
**Illness within 2 weeks (no as reference)**			
Yes	0.22	4.17	2.70–6.43**
**Perceived stress (low as reference)**			
High	0.27	8.60	5.09–14.54**
Moderate	0.231	3.99	2.54–6.27**
Constant	0.101	0.76	–

Adjusted for education level, occupation, work experience, contacted with an individual with confirmed or suspected COVID-19 infection, chronic disease, illness within 2 weeks, and perceived stress. *S.E.* standard error, *OR* odds ratio, *95%CI* 95% confidence interval

*: *P* < 0.05, **: *P* < 0.01

## Discussion

In the present study, the researchers explored the associated factors of poor sleep quality among community non-medical anti-epidemic workers in Wuhan, China, during the COVID-19 epidemic. The percentage of the participants who reported poor sleep quality was 46.20%, which was higher than the previously reported among the residents aged 15–69 years in China (35.74%) [23]. Poor sleep quality is a common problem in modern society [24], and during the COVID-19 epidemic, the community non-medical anti-epidemic workers were exposed to more risk factors that are related to sleep quality.

The present researchers found that the participants whose educational background was high school or technical secondary school were less likely to report poor sleep quality than those whose educational background was junior college or above. Moreover, the members of the police force were more likely to report poor sleep quality than volunteers. This finding is different from the based on a general population of Italian participants in a previous study [25]. A possible reason for this discrepancy is that the participants whose educational background was junior college or above and those who were members of the police force always undertake more anti-epidemic duties in the community during the COVID-19 epidemic, which would increase their negative emotions and stress, resulting in poor sleep quality. The present results suggested that participants who had contact with individuals with confirmed or suspected COVID-19 infection were more likely to report poor sleep quality. COVID-19 could transmit from human to human among close contacts [13]. Thus, contact with individuals with confirmed or suspected COVID-19 infection would obviously increase the risk of infection and psychological pressures [26], which leads to psychological problems and poor sleep quality. In terms of health status, the present researchers found that chronic disease and illness within 2 weeks were associated with poor sleep quality, which is similar to the findings from previous studies based on general populations [27, 28]. Chronic disease and illness within 2 weeks would definitely affect an individual’s quality of life, which has a close correlation with sleep quality [29].

Consistent with the previous findings from community residents [30], the present study manifested that perceived stress was an important factor related to sleep quality. Perceived stress is part of the individual’s psychological response after perceiving and evaluating a threatening stimulus around. When the individual under stress, the psychological threats or confusions will arise following the cognitive evaluation, lead to physical and psychological illnesses, such as poor sleep quality [31, 32].

The present study not only supplements previous studies, but also offers information about the community non-medical anti-epidemic workers. It examined multiple factors that are associated with sleep quality, such as demographic variables, health-related factors, social support, and perceived stress. Meanwhile, the questionnaire (PSQI) used in this study is reliable and can reflect individuals’ real sleep quality of the past month.

Nevertheless, the present study also has some limitations. First, although the sample size is not sufficient, with 15 communities were being included. Second, because the survey was conducted during the COVID-19 epidemic, the present researchers could not perform face-to-face interviews, and the data may not reflect the real situation of the participants. Third, the cause-effect relationship cannot be established because of the nature of cross-sectional design. Fourth, sleep quality can be influenced by occupation and life stresses, which no information on common variables were collected to assess the occupational impact.

## Conclusion

The present study revealed that community non-medical anti-epidemic workers who were members of the police force, workers with educational background of junior college or above, workers who had contact with individuals with confirmed or suspected COVID-19 infection, workers who had chronic disease(s), workers who had an illness within 2 weeks, and workers who had high or moderate perceived stress were more likely to report poor sleep quality. Therefore, the community non-medical anti-epidemic workers are required to follow appropriate personal-protection procedures and conduct psychological interventions through psychological lectures.

## Data Availability

The datasets used and/or analyzed during the current study are available from the corresponding author on reasonable request.

## Abbreviations

COVID-19: Coronavirus disease 2019
MSPSS: Multidimensional Scale of Perceived Social Support
PSS: Perceived Stress Scale
PSQI: Pittsburgh Sleep Quality Index
SD: Standard deviation
OR: Odds ratio
CI: Confidence interval
